# ESTclean: a cleaning tool for next-gen transcriptome shotgun sequencing

**DOI:** 10.1186/1471-2105-13-247

**Published:** 2012-09-26

**Authors:** Hongseok Tae, Dongsung Ryu, Suhas Sureshchandra, Jeong-Hyeon Choi

**Affiliations:** 1 Cancer Center, Department of Biostatistics, Georgia Health Sciences University, Augusta, GA 30912, USA; 2 The Center for Genomics and Bioinformatics, Indiana University, Bloomington, IN 47401, USA

## Abstract

**Background:**

With the advent of next-generation sequencing (NGS) technologies, full cDNA shotgun sequencing has become a major approach in the study of transcriptomes, and several different protocols in 454 sequencing have been invented. As each protocol uses its own short DNA tags or adapters attached to the ends of cDNA fragments for labeling or sequencing, different contaminants may lead to mis-assembly and inaccurate sequence products.

**Results:**

We have designed and implemented a new program for raw sequence cleaning in a graphical user interface and a batch script. The cleaning process consists of several modules including barcode trimming, sequencing adapter trimming, amplification primer trimming, poly-A tail trimming, vector screening and low quality region trimming. These modules can be combined based on various sequencing applications.

**Conclusions:**

ESTclean is a software package not only for cleaning cDNA sequences, but also for helping to develop sequencing protocols by providing summary tables and figures for sequencing quality control in a graphical user interface. It outperforms in cleaning read sequences from complicated sequencing protocols which use barcodes and multiple amplification primers.

## Background

Full cDNA shotgun sequencing is a major approach to finding whole transcriptomes and measuring gene expression. With the advent of next-generation sequencing (NGS) technologies [1] such as 454 (Roche) and Solexa (Illumina), NGS sequencing has become popular in the study of transcriptomes especially in non-model organisms because of its cost efficiency compared to Sanger. In addition, several protocols have been invented to apply NGS technologies and each protocol uses its own short DNA tags or adapters attached to the ends of DNA fragments for labeling or sequencing. Since NGS technologies eliminate bacterial cloning, library preparation is fast and cheap without vector contamination. However, a simple protocol for 454 transcriptome sequencing can make artifact sequences, e.g., concatenated amplification primers. This problem can be overcome by using several amplification steps each of which uses different primers [2].

In transcriptome sequencing projects, the quality of initial data greatly affects downstream analyses and removing contamination has become one of the most important steps. To remove contamination, several software tools are available, including VecScreen [3], Lucy [4], Cross_match [5], SeqClean [6], Figaro [7], and SeqTrim [8]. Although these programs have been used in many sequencing projects, most of them are not appropriate to detect the diverse contamination produced by several NGS-based protocols, especially those using two or more PCR amplification primers. None of them support new sequencing features such as barcodes or MIDs (Multiplex Identifiers), which are used to pool different samples. Many biologists also have difficulty using the programs due to complicated parameters, environment-specific operations and command line execution.

In this paper, we present a new program named ESTclean to clean raw sequences with seven modules that perform end sequence trimming, barcode trimming, sequencing adapter trimming, amplification primer trimming, poly-A tail trimming, vector screening and low quality region trimming. These modules can be combined based on various sequencing applications, e.g., parallel tagged sequencing [9]. ESTclean provides a GUI with a user-friendly environment to manage sequencing protocols and analysis pipelines. It also produces various summary tables and figures to aid quality control by showing trimming statistics for each module; identifying problematic reads with primer concatenation, wrongly oriented primers, and no barcodes; and assessing sequencing biases.

## Implementation

The most common sources of contamination in NGS-based ESTs are barcodes, sequencing adapters, and amplification primers. Barcodes or MIDs (Multiplex IDentifiers) are short DNA tags attached to the 5’ ends of reads in order to distinguish pooled samples. Sequencing adapters are attached to both ends of DNA fragments for cloning and sequencing. Although the 454 data processing software is supposed to trim sequencing adapters, 3’ sequencing adapters often remain depending on the software version and fragment size. Amplification primers are attached to both ends of cDNAs to prepare cDNA libraries before fragmentation. These primers are often concatenated to each other in badly designed sequencing protocols.

**Figure 1:**
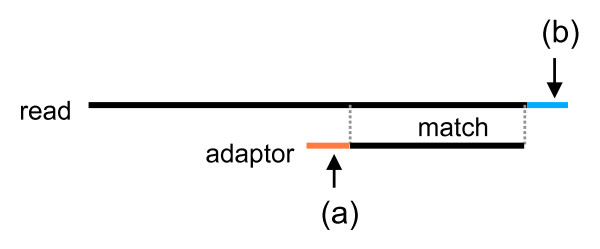
**Amplification primer trimming.** When a primer matches partially to the end of a read, ESTclean checks the minimum percent identity and length of the match and the minimum number of unaligned bases in the primer (**a**) and read (**b**).

A semi-global algorithm is implemented to search barcodes from the 5’ end of a read sequence. If the number of mismatches and indels between a barcode and a read exceeds allowable errors, then the read is discarded. Otherwise the barcode is trimmed and used to separate reads by sample. ESTclean uses BLAST [10] to search sequencing adapters and amplification primers against reads. Since BLAST cannot align the ends of reads with sequencing errors, we extend the alignment using the banded Needleman-Wunsch algorithm [11] and allow a small number of unaligned bases at the ends. Primers and adapters can match to the middle or ends of a read. Therefore we need a different criterion for such cases. If primers and adapters match to the middle of a read, then they should match near perfectly. In this case, we use a minimum alignment length and percent identity. However, if they match to the ends of a read, they can match partially. In such cases we use three parameters: the minimum percent identity of an alignment and the minimum numbers of unaligned bases in the primer and read (Figure 1).

**Figure 2:**
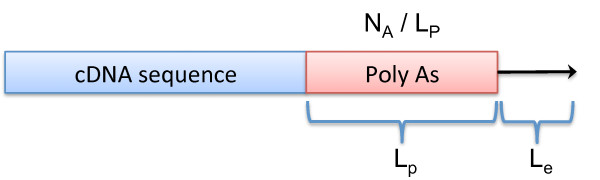
**Poly A tail trimming.** A poly A tail is recognized by the length (*L*_*p*_) of the tail, the ratio of the number (*N*_*A*_) of As to *L*_*p*_, and the length (*L*_*e*_) of the 3’ end.

Poly-A, consisting of multiple adenosines, is a stretch of a eukaryotic messenger RNA (mRNA) and is important for translation and stability of the mRNA. The sequence of cDNAs contain poly-A tail or poly-T head sequences because cDNA sequencing uses reverse transcription polymerase chain reaction (RT-PCR) with amplification primers that have poly-As to make cDNA libraries. However, because amplification primers do not contain an entire poly-A tail, we need to trim As and Ts before 3’ and after 5’ amplification primers respectively. The starting site of poly-As should be a certain number of bases from the end (Figure 2). We search for A and T in the 3’ and 5’ ends respectively and expand them toward the middle of a sequence as long as the fraction of As or Ts is greater than a cutoff. If those regions are greater than or equal to the minimum length of poly-As, then they are trimmed out.

Although NGS-based cDNA sequencing does not use vectors for amplification, ESTclean has a module to screen known vectors using VecScreen [3]. ESTclean also has a module to modify SFF files to set a clean region for each read if users have SFF tools. Discarded read sequences from any steps can be collected and saved as a FASTA file and analyzed using BLAST with a user-provided sequence database.

**Figure 3:**
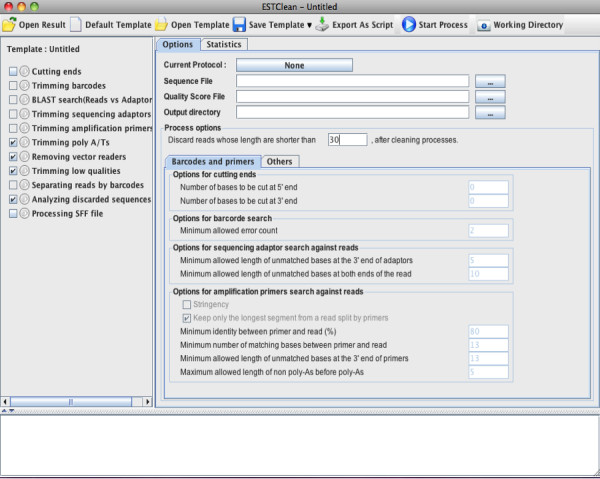
**ESTclean screenshot.** The left panel displays the steps and progress in a cleaning process. On the right panel, the options tab is used for specifying a sequence and quality score files, an output directory, and parameters for all cleaning modules. The statistics tab shows various statistics of cleaning for quality control. The bottom panel displays messages and errors during cleaning processes.

**Figure 4:**
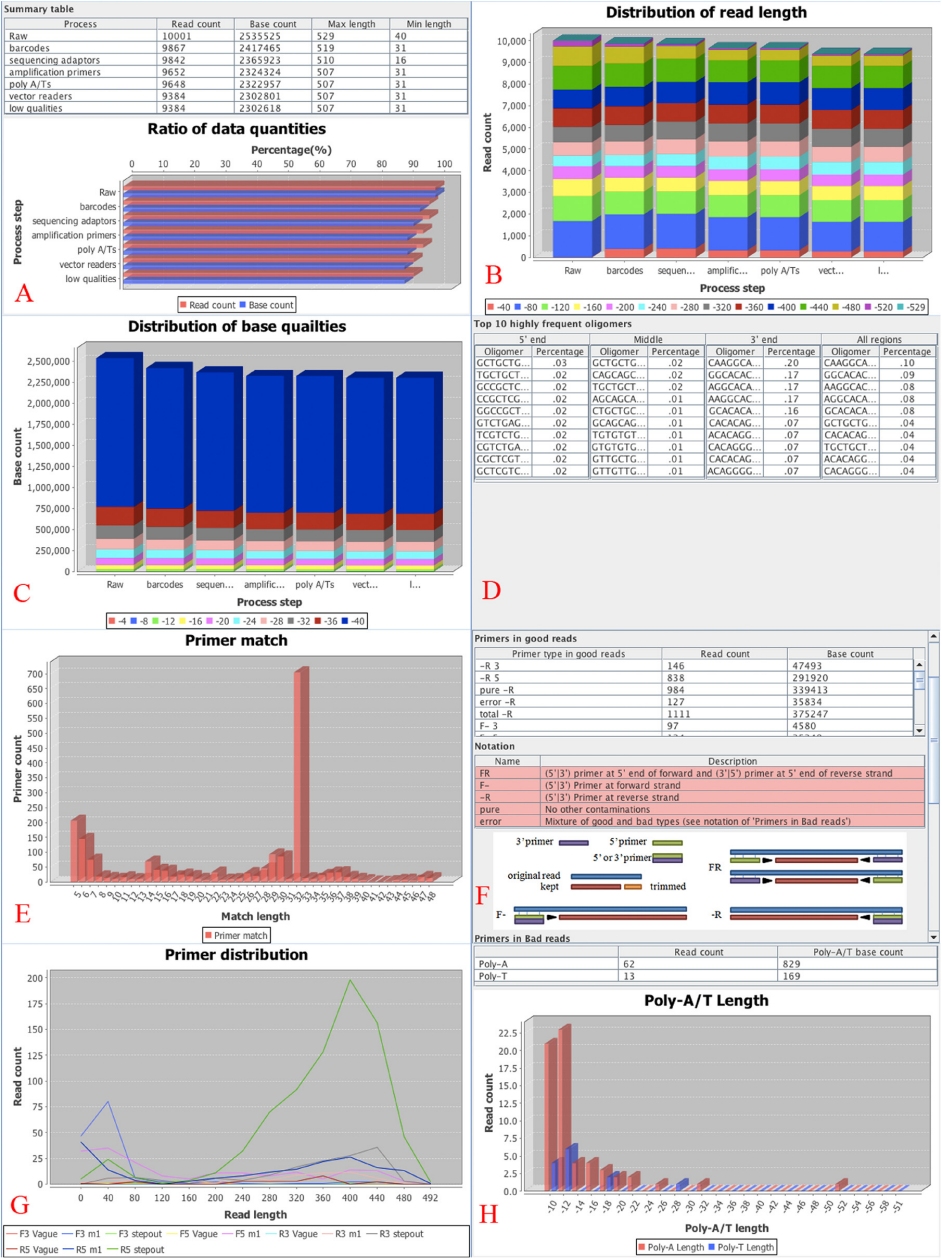
**Summary tables and figures.** For validation of final products, several charts and tables are provided in order to display statistical information of trimming results. **A**: The numbers of reads and bases, and minimum and maximum read lengths for each cleaning step. **B**: The distribution of read lengths for each cleaning step. **C**: The distribution of quality scores for each cleaning step. **D**: The percentage of top 30 k-mers in cleaned sequences. **E**: The histogram of primer matches. **F**: The number of good and bad reads in terms of primer combinations. **G**: The number of primers identified at each base position. **H**: The histogram of lengths of trimmed poly A tails and T heads.

The main executable scripts of this package have been developed in PERL and the user-friendly GUI has been developed in JAVA (Additional files 1, 2, 3). As shown in Figure 3, the GUI enables users to set sequencing protocols, input their own sequences to be trimmed, set parameters for each module, and choose modules to run. To set a sequencing protocol, users input the sequences of amplification primers, sequencing adapters and barcodes. Sequencing protocols can be imported and exported in the FASTA format. When the cleaning procedure starts, the program puts the selected modules into a task queue and validates the parameters. The left panel of the interface displays the running status. After cleaning, ESTclean provides several charts and tables for summary (Figure 4), which are very important for quality control. The tables and charts can be stored into a project file for future use. User-defined parameters are stored in a template and can be used in future projects.

**Figure 5:**
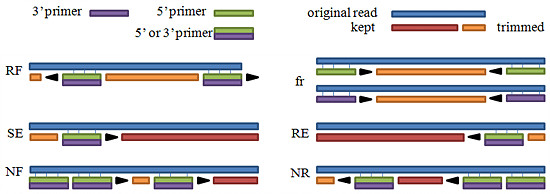
**Erroneous read types.RF**: reverse and forward matched primers in the 5’ and 3’ ends respectively; **fr**: forward and reverse matches of the same primer; **SF**: forward match in the 5’ end but with unaligned bases before it; **RE**: reverse match in the 3’ end but with unaligned bases after it; **NF**: multiple forward matches; **NR**: multiple reverse matches.

One of the unique features of ESTclean is to show what kind of sequencing errors are present in sequencing data. Error-free reads can have PCR amplification primers forward matched in the 5’ end and/or reversely matched in the 3’ end. However, as shown in Figure 5, erroneous reads have reverse and forward matched primers in the 5’ and 3’ ends respectively (RF); forward and reverse matches of the same primer (fr); forward match in the 5’ end but with unaligned bases before it (SF); reverse match in the 3’ end but with unaligned bases after it (RE); multiple forward matches (NF); and multiple reverse matches (NR).

## Results and discussion

**Figure 6:**
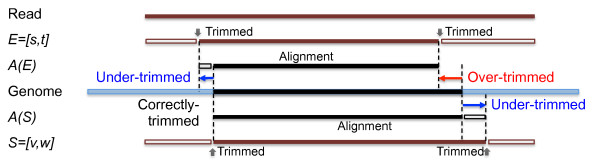
**Evaluation method.** Mapping results, *A*(*E*) and *A*(*S*), by GMAP for reads, *E* and *S*, cleaned by SeqClean and ESTclean respectively are evaluated to decide whether the reads are over- or under-trimmed. At the 5’ end, while SeqClean performs correct trimming, the read from ESTclean is under-trimmed as its 5’ end is not aligned to the genome. At the 3’ end, ESTclean over-trims while SeqClean under-trims because the latter has unaligned bases and the trimmed region of the former is real (aligned).

To demonstrate the performance of ESTclean, we used a real 454 sequencing run for *Drosophila melanogaster* and compared to SeqClean [6]. SeqClean is a tool that performs automated trimming and validation of ESTs or other DNA sequences by screening various contaminants, low quality and low complexity sequences. It utilizes BLAST [10] to remove any sequence highly similar to a given list of vectors, adapters, primers or linker sequences that are located within 30% of total EST from the 3’ or 5’ end of the sequences. The raw sequence reads were cleaned using SeqClean with input.fna -c 10 -l 30 -v barcode_adapter_primer -o output.seqclean and using ESTclean in GUI with the default parameters and non-stringent amplification primer and poly-A search (BLAST version 2.2.20). We used GMAP (version 2011-11-14) [12] with -D dmelchrs -d dmelchrs -f psl input.fna output.psl to map reads cleaned by ESTclean and SeqClean, respectively, to the *D. melanogaster* genome (FlyBase Release 5.13). Since a cleaned read is defined as an interval, let a cleaned read by ESTclean and SeqClean be *E* = *st* and *S* = *vw*, respectively. We discarded reads mapped to multiple locations in the genome. Let the alignment positions in the genome for a cleaned read by ESTclean be *A*(*E*) = *s*^*′*^*t*^*′*^and *A*(*S*) = *v*^*′*^*w*^*′*^. We then identified the best position between both alignments in 5’ and 3’ ends respectively, i.e., *A*(*B*) = *min*(*s*^*′*^*v*^*′*^),*max*(*t*^*′*^*w*^*′*^)]. If a cleaned read is not fully aligned to the genome, then the read is under-trimmed. Otherwise, it is over-trimmed if its alignment position is not the best one, e.g., *s*^*′*^ ≠ *min*(*s*^*′*^*v*^*′*^) for *E* (Figure 6).

**Figure 7:**
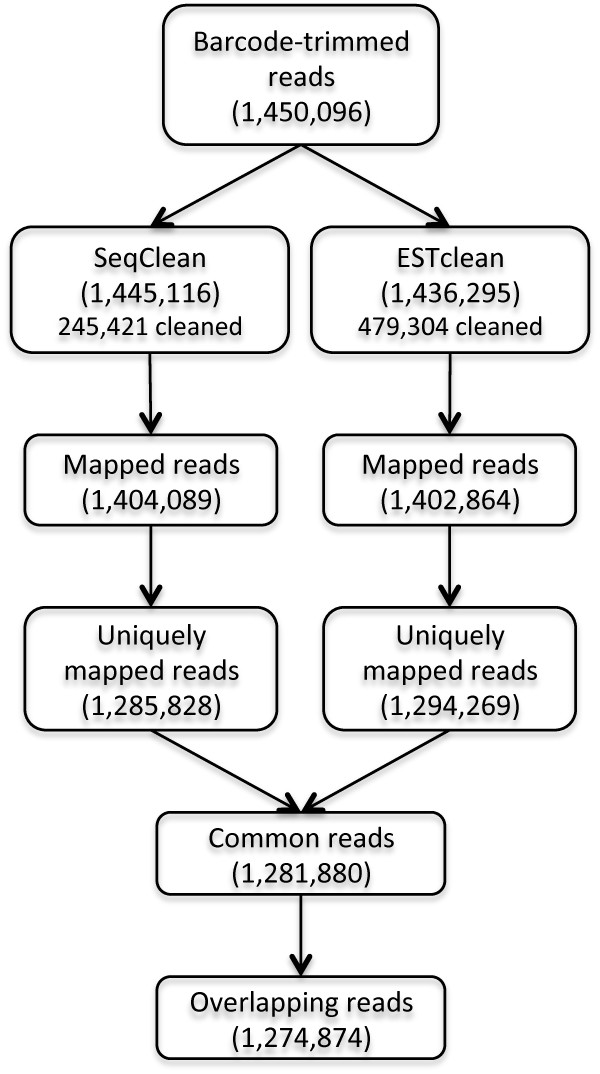
**Experiment workflow.** Since SeqClean cannot trim a barcode, barcode-trimmed reads by ESTclean were used as input data. The cleaned reads by SeqClean and ESTclean were mapped to the reference genome using GMAP. We filtered out multiply mapped reads and non-overlapping reads by at least 40bp in the genome. Finally 1,290,547 reads were used for evaluation.

Of 1,453,938 reads, SeqClean and ESTclean left over 1,449,125 and 1,436,295 reads respectively after cleaning. Out of these, 242,683 and 1,436,295 reads were cleaned by at least 1bp. Surprisingly, SeqClean cannot trim a barcode sequence in the 5’ end although this sequencing protocol has a barcode, meaning that sequence reads with no barcode are artifacts. Therefore, we decided to use 1,450,096 reads that were barcode trimmed by ESTclean (Figure 7). SeqClean trimmed 245,421 reads while ESTclean trimmed 479,304 reads. Of 1,445,116 and 1,436,295 reads left over by the programs, GMAP mapped 1,404,089 and 1,402,864 reads to the reference genome. ESTclean had more uniquely mapped reads while SeqClean had more multiply mapped reads. Of 1,281,880 reads that were mapped uniquely and commonly by both programs, 1,274,874 reads which overlap more than 40bp in the genome were evaluated (Figure 7).

**Figure 8:**
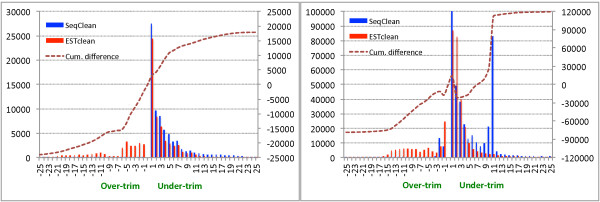
**Histogram of over- and under-trimmed lengths in the 5’ (left) and 3’ (right) ends.** The positive and negative X axes represent over- and under-trimming respectively. The dotted red line represents the cumulative difference in the number of over- and under-trimmed reads between ESTclean and SeqClean.

SeqClean and ESTclean over-trimmed 25,347 and 127,895 reads respectively while they under-trimmed 486,981 and 346,901 reads (Table 1). It is interesting that ESTclean outperformed SeqClean in terms of under-trimming, while SeqClean outperformed ESTclean in terms of over-trimming. Out of the under-trimmed reads, 338,264 and 181,588 were not trimmed at all. Figure 8 shows histograms of the lengths over- and under-trimmed by SeqClean and ESTclean in the 5’ and 3’ ends. The cumulative difference between ESTclean and SeqClean for given trimmed lengths shows the tendency of both programs. It is interesting that SeqClean did not trim many reads about 11 bp in the 3’ end, which results from the sequencing adapter.

**Table 1:** Evaluation result

**Strand**	**5’**		**3’**	
Program	SeqClean	ESTclean	SeqClean	ESTclean
Under-trimmed	79,369	61,138	407,612	285,763
Over-trimmed	739	25,055	24,608	102,840

However, over-trimming may be correct trimming without knowing reference sequences. What would happen if the bases next to a sequence read in a genomic location would be the same as the first bases of sequencing adapters, amplification primers, or poly A tails? For example, if a sequence read ACGTcaat comes from ACGTCGGA of a genome and the lower bases in the sequence read is a amplification primer, the caat should be cleaned by ESTclean. However GMAP can align the raw read until base c and perfect cleaning of caat is evaluated as over-trimming by 1 bp. We expanded this observation for all of over-trimmed reads but not trimmed due to low quality scores. Additional file 4 shows the over-trimmed subsequences by ESTclean in the 5’ and 3’ ends. Most of those sequences are part of sequencing adapters and amplification primers, especially poly A tails. To confirm this, we extracted trimmed subsequences of length 6 bases including an over-trimmed region and investigated these 6-mers. Indeed, almost all are part of sequencing adapters and poly A tails: 18,759 (100%) and 68,999 (92%) of reads over-trimmed in the 5’ and 3’ ends, respectively (Additional file 5).

## Conclusions

Since incomplete cleaning of EST sequences leads to incorrect downstream analyses such as mis-assembly and inaccurate biological interpretation. It has become one of the important tasks in transcriptome sequencing. ESTclean has been developed to remove the different kinds of contaminants from raw sequences. It not only provides trimming and screening modules, but also useful and user-friendly features including project management and quality control of sequencing protocols and raw sequences. It can also generate a script to execute trimming modules in command line environment in order to support automated pipeline of sequence assembly processes. We compared the performance of ESTclean with SeqClean for a real sequencing run for *Drosophila melanogaster*. ESTclean outperformed SeqClean in terms of the numbers of under-trimmed reads and bases. Although ESTclean has more over-trimmed reads in this experiment, it resulted from correct trimming without knowing reference sequences.

## Availability and requirements

**Project Name:** ESTclean 

**Project home page:**http://sourceforge.net/projects/estclean/

**Operating system(s):** Platform independent 

**Programming language:** Perl (v5.0 or later), Java (v1.5.0 or later) 

**Other requirements:** BLAST (v2.2.9 or later) (ftp://ftp.ncbi.nlm.nih.gov/blast/executables/LATEST) 

**License:** GNU GPL 

**Any restrictions to use by non-academic users:** license needed

## Competing interests

The authors declare that they have no competing interests.

## Authors’ contributions

JHC conceived the software function and architecture. JHC and HT implemented Perl and Java codes respectively. DR conducted the experiment to compare ESTclean to SeqClean using a 454 sequencing run for *Drosophila melanogaster*. SS tested the software with the real datasets and pointed out bugs and improvements. All authors have contributed to, read, and approved the final manuscript.

## Supplementary Material

Additional file 1 Program. Click here for file

Additional file 2 Manual. Click here for file

Additional file 3 Sample data. Click here for file

Additional file 4 Over-trimmed subsequences by ESTclean in the 5’ and 3’ end. Click here for file

Additional file 5 Distribution of 6-mers in over-trimmed sequences. Click here for file
