# Supplementary material for: ESTclean: a cleaning tool for next-gen transcriptome shotgun sequencing
Source: BMC Bioinformatics. 2012 Sep 26;13:247. doi: 10.1186/1471-2105-13-247 (PMC3630001; doi:10.1186/1471-2105-13-247)
Supplement: Additional file 4 — Over-trimmed subsequences by ESTclean in the 5’ and 3’ end. [file 1471-2105-13-247-S4.pdf]

ESTclean

1. Overview

As the sequencing process consists of several complicated steps, quality of initial data greatly affects downstream analyses. Thus issues related to data quality have become one of the most important challenges in sequencing projects. ESTclean is a software package to clean raw sequences by removing amplification primers, sequencing adaptors and vectors.

Cleaning process consists of six modules including barcode trimming, sequencing adaptor trimming, amplification adaptor trimming, poly-A tail trimming, vector screening and low quality region trimming. Main programs of this package have been written in PERL, and a window interface with a user-friendly working environment has been developed in JAVA.

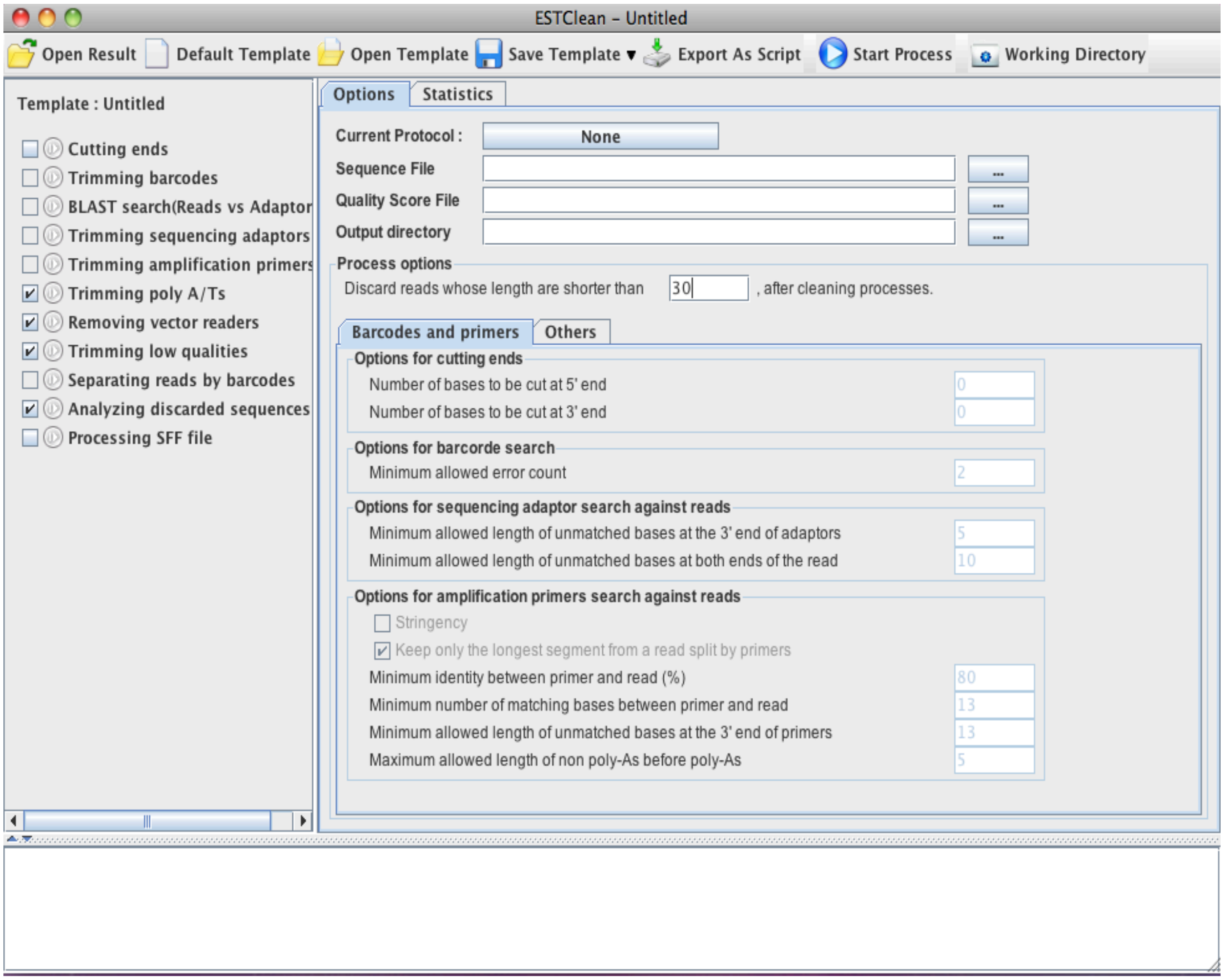

Key Features

- Barcodes trimming
- Sequencing adaptors trimming
- Amplification primers trimming
- Poly-A tails trimming
- Vector removal
- Low quality regions trimming
- Separation of reads according to their barcodes
- Analysis of discarded sequences
- Processing SFF files

2. Requirements

PERL v5.0 or above: http://www.perl.org/get.html
JAVA v1.5.0 or above: http://java.sun.com/javase/downloads
BLAST: ftp://ftp.ncbi.nih.gov/blast/executables/LATEST
Sfffile: (optional) for cleaned SFF output.

3. How to start?

To start the program, users can simply type the following scripts:

```
>./estclean.sh (for Unix, 'estclean.bat' for Windows) or
>java –Xmx1g -jar estclean.jar
```

The first time the program starts, users need to set the working directory to store the environment files. Cleaning PERL programs and primer sequences of ESTclean are stored in this directory. If a new version of the software has been downloaded and same working directories were used for previous versions, make sure the bin directory in the working directory is deleted, to remove the old environment files.

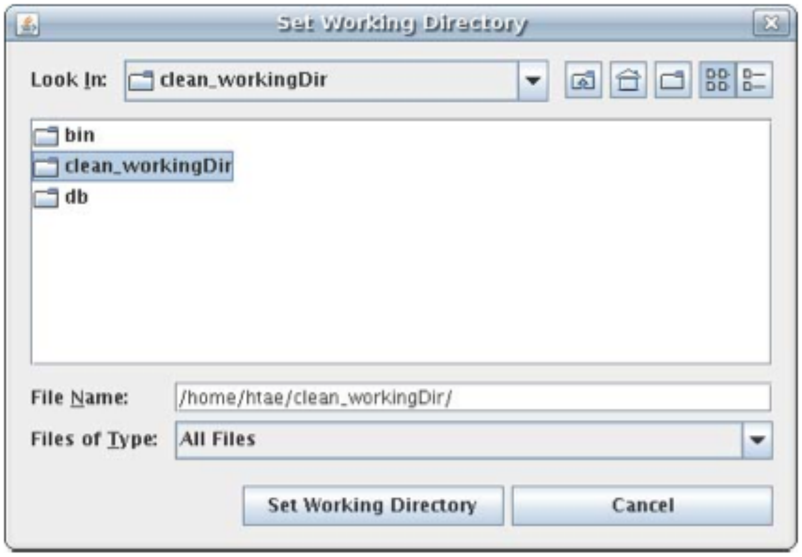

4. Trimming steps

1) Barcodes trimming

Barcodes or MIDs (Multiplier Identifiers) are short DNA tags to identify which samples they are from. They are attached to 5' ends of reads during library preparation. Since barcodes are not a part of the original DNA sequences, they should be removed before downstream analyses. In this step, the barcodes are screened and removed from the read sequences.

2) Sequencing adaptors trimming

Sequencing adaptors are attached to both ends of DNA fragments in order to aid cloning and sequencing. Although the data processing software in a sequencing system such as 454 is supposed to trim sequencing adaptors, 3' sequencing adaptors still remain attached to the DNA. Also a sequencing protocol could attach additional adaptors to the DNA fragments. Our program supports trimming various kinds of adaptors by utilizing BLAST.

3) Amplification primers trimming

Before fragmentation of cDNAs, they are amplified with two amplification adaptors attached to their both ends in order to obtain enough coverage. As they are often concatenated to each other, their contamination is more complicated than sequencing adaptors.

4) Poly-A trimming

The poly-A tail, which consists of multiple adenosines, is a stretch of a eukaryotic messenger RNA (mRNA) and is important for translation and stability of the mRNA. mRNA sequences are analyzed by sequencing their cDNAs generated by RT-PCR. Thus the cDNA also contains poly-A tail or poly-T head sequences. Poly-A/Ts need to be removed from read sequences after trimming primers.

5) Vector removing

In the Sanger sequencing method, DNA fragments are usually inserted into diverse cloning vectors such as plasmid, phage, cosmid, BAC and YAC, and the resulting sequences may contain parts of the vectors.

6) Low quality regions trimming

ESTclean removes low quality bases from reads in order to improve assembly accuracy.

7) Separation of reads according to barcodes

If reads are attached to several different barcodes, ESTclean separates reads based on their barcodes.

8) Analysis of discarded sequences

ESTclean provides a method to analyze the reads discarded in previous trimming steps. Users can compare them with other sequence data using the BLAST.

9) Process of SFF files

If an executable 'sfffile' file is available, ESTclean trims SFF format files.

5. Template management

Users can trim the contaminants and low quality sequences by executing ESTclean with default parameters, and modify the parameters for the detailed procedures. The modified parameters can be saved in to the template and reloaded from it.

6. Features of the main window

1) Toolbar

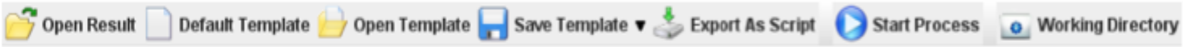

- Open Result: It opens a result file created by ESTclean.
- Default Template: It sets all parameters with default values.
- Open Template: It reloads parameters from a template.
- Save Template: It saves parameters into a template.
- Export As Script: It creates a PERL script to run the ESTclean program from the command line with given parameters.
- Start Process: It starts the trimming process with given parameters. When the process starts, the label turns to 'Stop Process' and if clicked the process stops.
- Working Directory: User can change the working directory.

2) Procedure states

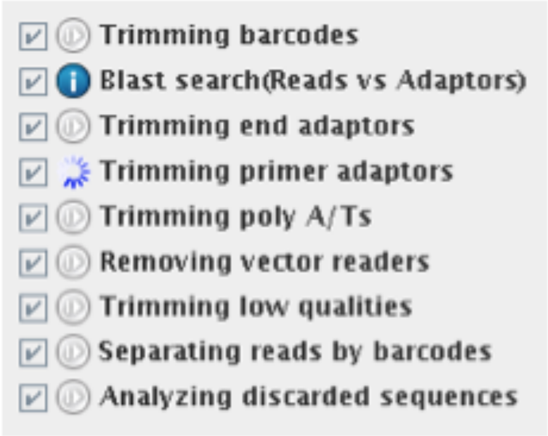

This frame shows the progress of the trimming steps. User can check skipped and completed steps as well as a running step.

3) Parameters frame

The detailed parameters can be set in the parameters frame. The top two textboxes take the sequence file and the quality score file as the input. With the other parameters, ESTclean screens contaminant sequences and removes them

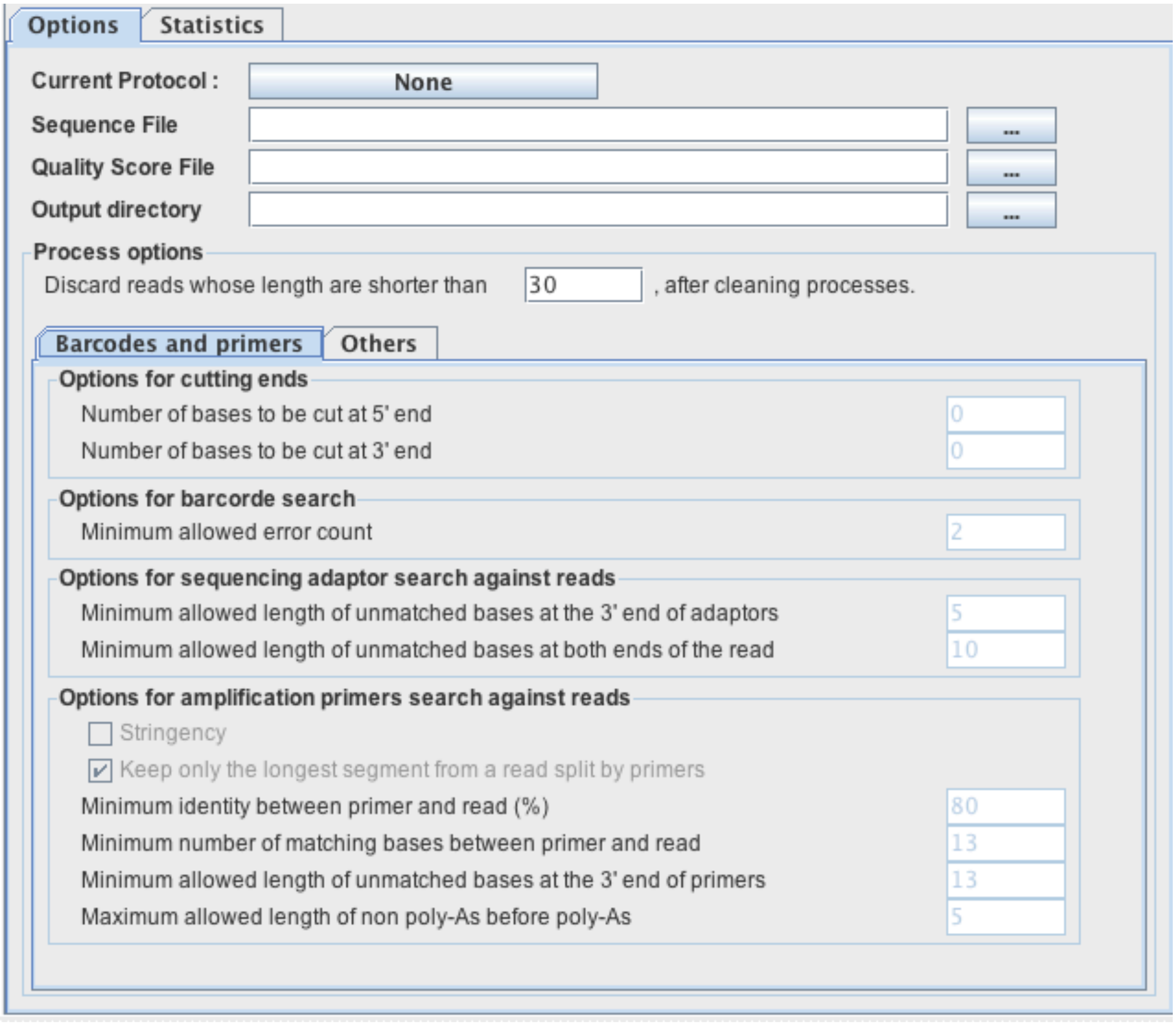

The above snapshot shows the parameters for barcodes and primers.

Other parameters (for poly-As, VecScreen and BLAST) are shown below.

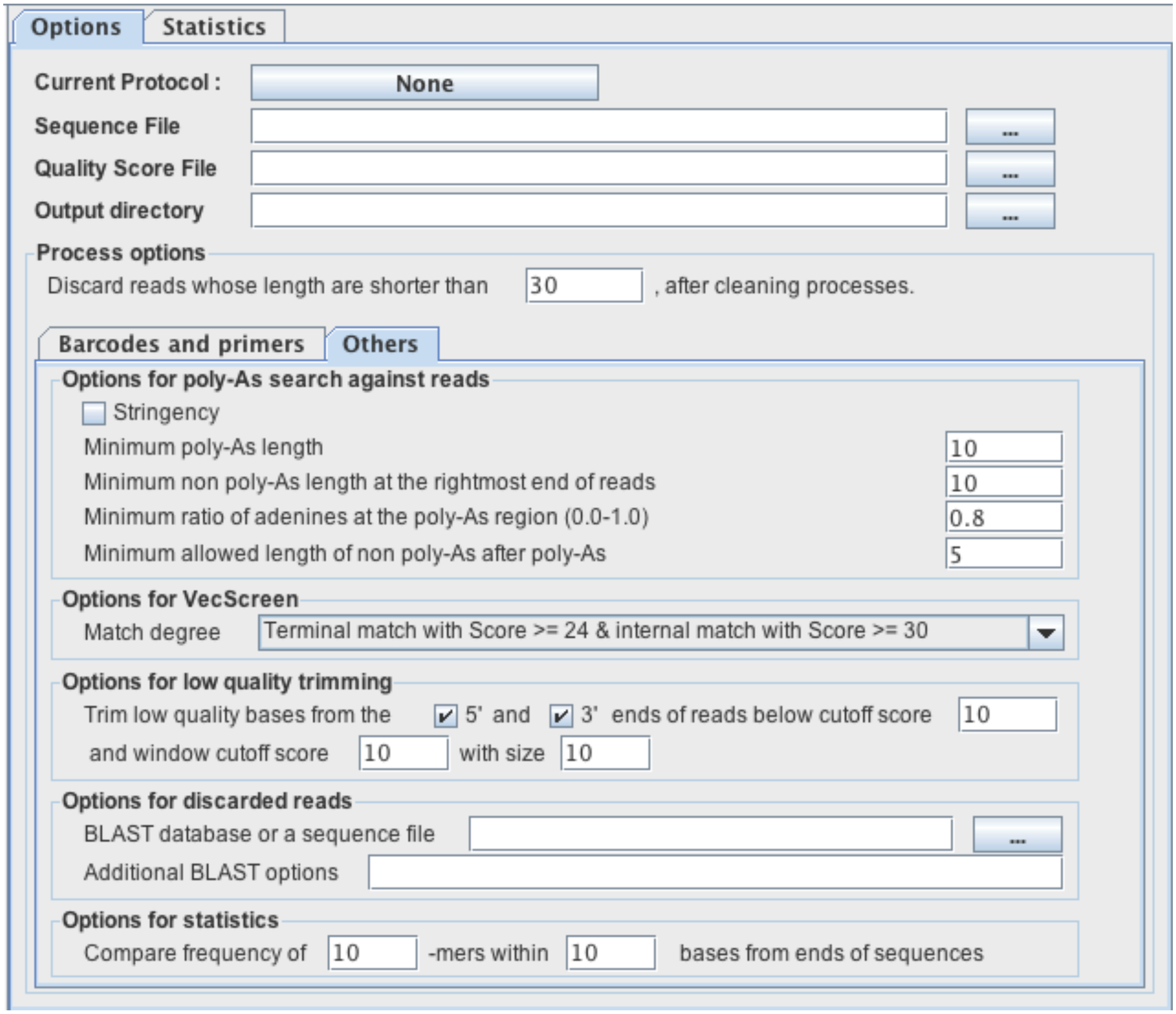

4) Message frame

The messages from the several steps are printed in the message frame.

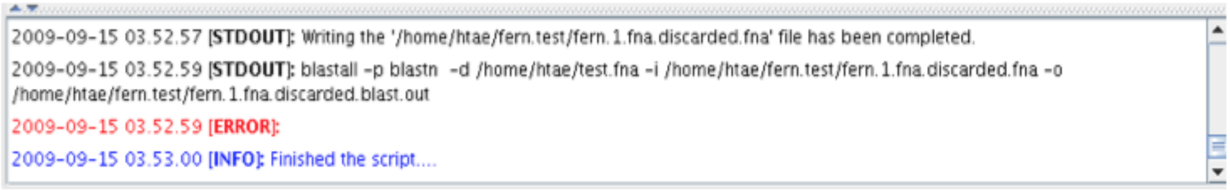

7. Parameters for each step.

1) **Global parameters**

(a) *Sequence File*: A file containing read sequences.
(b) *Quality Score File*: A file containing quality scores.
(c*) Discard reads whose lengths are shorter than 'x', after cleaning processes*: During each step, the reads whose lengths are shorter than 'x' are discarded.
(d) *Checkbox for strong search*: If this checkbox is selected, ESTclean finds contaminant sequences under stringent conditions.
(e) *Current Protocol*: It shows the name of the current protocol, which contains the user-defined sequences of barcodes, sequencing adaptors and amplification primers. User can change the protocol by clicking the 'Change Protocol' button and modifying the parameters of a dialog window (See 8. Protocol dialog).

2) **Options for cutting ends**

This includes options for number of bases to be cut at 5' end and 3'end. If the input file has been generated from sff file, then the sequences include 4 base key sequences, which need to be cut at its 5'end . In such cases, set number of bases to be cut at 5' end as 4.

3) **Options for barcode search against read**

(a) *Minimum allowed error count*: It is the minimum number of mismatches or gaps allowed in the alignment between a barcode and a read. If the number of mismatches is over this error count, the sequence is not considered a barcode.

4) **Options for sequencing adaptor search against read**

(a) *Minimum allowed length of unmatched bases at the 3' end of the adaptor*: If the length of unmatched bases at the 3' end of the adaptor is over this value, the BLAST hit is not considered a match of the read and the adaptor. (shown as 'a' in figure)
(b) *Minimal allowed length of unmatched bases on both ends of reads*: If the length of unmatched bases at the end of reads is over this value, the BLAST hit is not considered a match of the read and the adaptor. (shown as 'b' in figure)

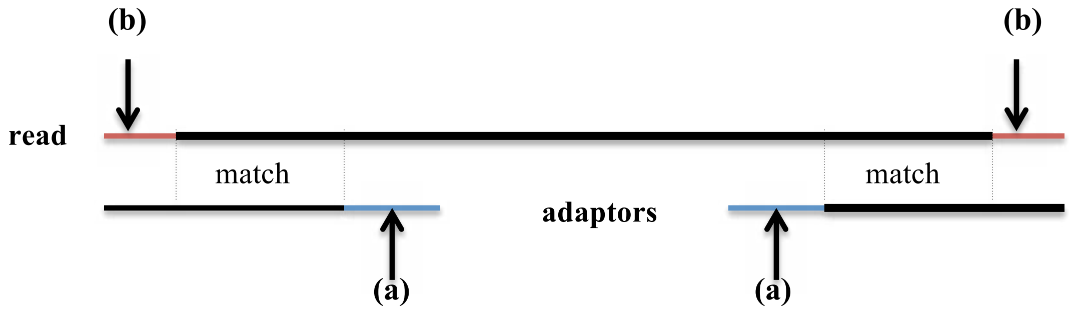

5) **Options for primer search against read**

(a) *Retain best segments of reads only*: When the primer sequences are detected in the middle of a read sequence, two or more segments may be produced from the trimming step. If this checkbox is selected, only the longest segment of the read is retained and the rest are discarded.
(b) *Minimum identity between primer and read*: If percentage identity between a primer sequence and a read sequence is over this value, the match region is considered a primer sequence.
(c) *Minimum match length between primer and read*: If the length of a match between a primer sequence and a read sequence is over this value, the region is considered a primer sequence.

6) **Options for poly-A search against read**

(a) *Minimum poly-A length*: The poly-As whose lengths are over this value are removed.
(b) *Minimum non poly-A length at the rightmost end of reads*: If there is non poly-A sequence at the rightmost end of a read and its length is over this value, even though there is poly-A sequence at the 3' of the read, it would not be removed. (ex. ATAC......TCAAAATAAAA**CGT**)
(c) *Minimum ratio of adenines at the poly-A region (0.0-1.0):* If the ratio is under this value, the poly-A sequence is not removed.

7) **Options for VecScreen**

VecScreen is a system developed by NCBI for quickly identifying segments of a nucleic acid sequence that may be of vector origin.

(a) *Match degree*: Vector contamination usually occurs at the beginning or end of a sequence; therefore, different criteria are applied for terminal and internal matches.
Strong Match to Vector
(Expect 1 random match in 1,000,000 queries of length 350 kb.)
Terminal match with Score ≥ 24 and Internal match with Score ≥ 30.

Moderate Match to Vector
(Expect 1 random match in 1,000 queries of length 350 kb.)
Terminal match with Score 19 to 23 and Internal match with Score 25 to 29

Weak Match to Vector
(Expect 1 random match in 40 queries of length 350 kb.)
Terminal match with Score 16 to 18 and Internal match with Score 23 to 24.

8) **<u>Options for low quality trimming</u>**

ESTclean screens the low quality bases from 5' and 3' ends of reads and removes them. Two steps are applied to screens the bases. First, ESTclean searches for the low quality bases from the end positions and stops the process at a base whose quality is over the cutoff score. Then it uses a window with given size to search for the low quality bases from the end positions. When the average quality score of bases in the window falls below the cutoff score, the region is removed and the window moves from the end position. This process also stops when the average quality score of bases in the window is over the cutoff score.

9) **<u>Options for discarded reads</u>**

(a) *BLAST database or a sequence file*: The discarded reads would be compared with given data using the BLAST program. If a sequence file is selected, ESTclean executes *formatdb* for the given file.
(b) *Additional BLAST options*: Users can add BLAST options in this textbox.

8. Protocol dialog.

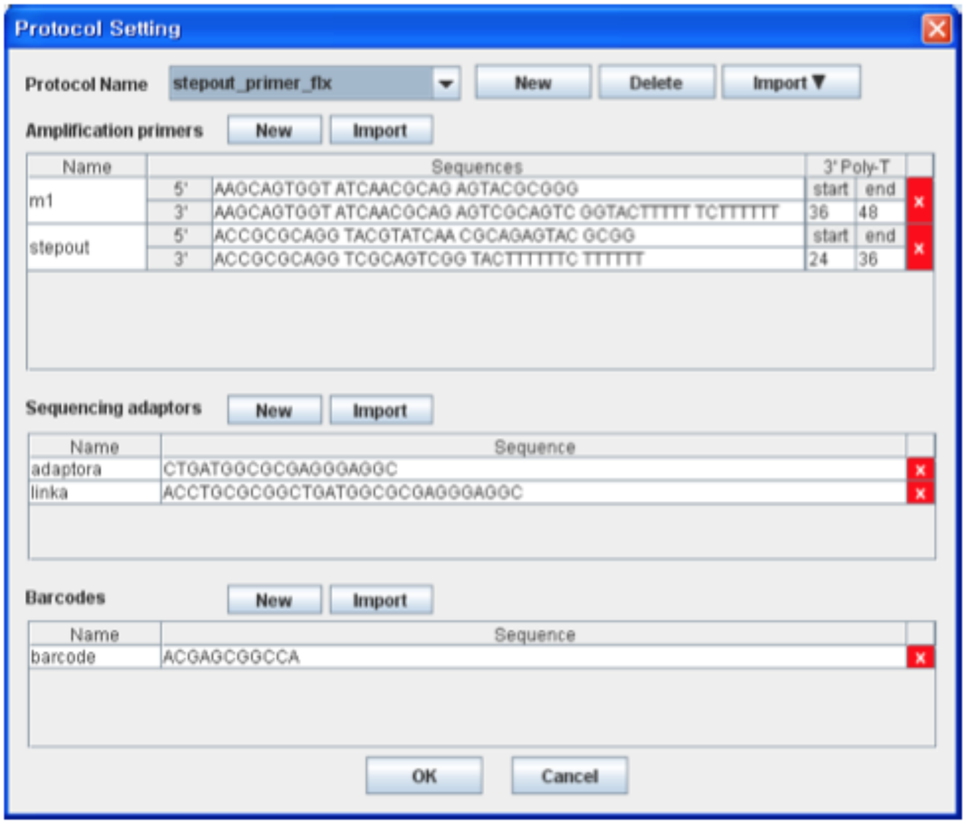

Sequences of primers, sequencing adaptors and barcodes inserted in this dialog are used to trim the reads. If there is poly-T sequence at the end of 3'primer, the region can be designated by a user. The sequences in the dialog are managed as a set called a protocol. The sequences of other protocols or files can be imported into the selected protocol.

- Protocol
  (a) New: It creates a new protocol.
  (b) Delete: It deletes a current protocol.
  (c) Import: It imports sequences w
  From protocol: It imports sequences from another protocol
  From file: It imports sequences from files.

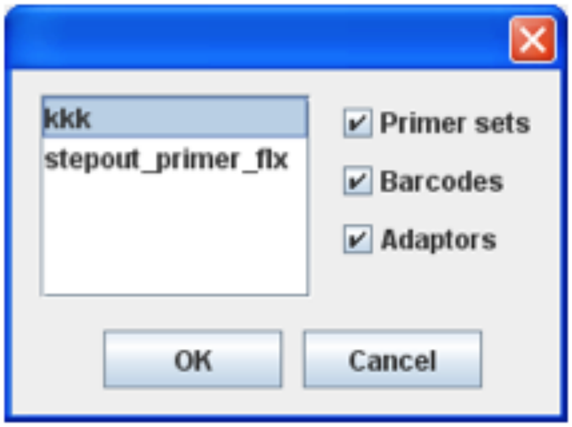

The following is a set of sequences imported from a file.

m1 and stepout (ending with .5 and .3) are amplification primers while A, B and linkb are sequencing adaptors.

```
>m1.5
AAGCAGTGGTATCAACGCAGAGTACGCGGG

>m1.3 36 48
AAGCAGTGGTATCAACGCAGAGTCGCAGTCGGTACTTTTTTCTTTTTT

>stepout.5
ACGAGCGGCCAGTATCAACGCAGAGTACGCGG

>stepout.3 24 36
ACGAGCGGCCACGCAGTCGGTACTTTTTTCTTTTTT

>A
CTGAGTCGGAGACACGCAGGGATGAGATGG

>B
CTGAGACTGCCAAGGCACACAGGGGATAGG

>linkb
TGGCCGCTCGTCTGAGACTGCCAAGGCACACAGGGGATAGG

>barcode
CCGCGCAGGT
```
