# Supplementary material for: ESTclean: a cleaning tool for next-gen transcriptome shotgun sequencing
Source: BMC Bioinformatics. 2012 Sep 26;13:247. doi: 10.1186/1471-2105-13-247 (PMC3630001; doi:10.1186/1471-2105-13-247)
Supplement: Additional file 5 — Distribution of 6-mers in over-trimmed sequences. [file 1471-2105-13-247-S5.zip › sample/README.docx]

**Running ESTclean on sample file**

To test run ESTclean, we have provided sample files on the project website. On unzipping the file estclean_sample.zip, a directory called sample is generated with input file sample.fna, quality file sample.qual and protocol file stepout_titanium. The protocol file contains amplification primers, adapters and barcodes. If needed, users can create the required individual barcode files, amplification primers file and adapters file and use those files to create a protocol.

- Once the GUI is ready to be used, the working directory needs to be set. If a new version of the software has been downloaded and same working directories were used for previous versions, make sure the bin directory in the working directory is deleted, to remove the old environment files. The working directory can be changed by clicking on *Working Directory* in the toolbar.
- By default, below the toolbar, *Options* tab is selected. Here, click the button next to *Current Protocol*. Click on *New* to provide a protocol name and update the protocol information. The information can be updated by importing a file. For this, click on *Import+* followed by *From files*. In this case the file stepout_titanium has to be imported. For subsequent use, users can import a protocol using *Import+* followed by *From protocols.* Also if users need to upload or create individual barcode files, sequencing adapter files or amplification primer files, the dialog box has provisions to do that. Just click on *New* or *Import* in each category. Once complete, click *OK* to save the protocol.
- Next, upload the input sequence file next to the entry *Sequence file.* If the quality file has the same name as the input fna file, the quality file gets automatically uploaded in the *Quality Score File.* If not, upload the quality score file. However, the software can run without a quality score file.
- Set the output directory where all the intermediate cleaned files, log files, final output file and the project gets saved.
- Depending on the inputs provided, jobs get checked on procedures frame on the left side. On the right side, the tab *Barcodes and primers* allows users to choose the options for cutting ends, barcode search, adapter search, stringency and amplification primer search. This can be altered according to user’s convenience. **N.B. If the sequence file has been generated from sff file, then the sequences include 4 base key sequences, which need to be cut at its 5’ end. In such cases, set *Number of bases to be cut at 5’end* as 4*.***On choosing *Others* tab, user can alter parameters for ploy-A searches, VecScreen, low quality trimming and blast options for analyzing discarded reads.
- Once all parameters have been set, you might want to save these settings by clicking on *Save Template* in the toolbar. You can retrieve previously saved settings by clicking on *Open Template* option in the toolbar.
- To run the software, click on *Start Process* button in the toolbar. The log frame at the bottom gets updated with the job status. Also changes can be seen on the procedures frame on the left as jobs progress. Clicking on *Statistics* tab next to *Options* tab (below toolbar) lets user visualize graphs for read summary, read lengths, quality scores and K-mer rates. Once the job completes, the project file gets saved in the output directory. This project file can later be loaded using *Open Result* option in the toolbar.
